# Supplementary material for: Periosteal skeletal stem cells can migrate into the bone marrow and support hematopoiesis after injury
Source: bioRxiv. 2023 Jan 13:2023.01.12.523842. Preprint. [Version 1] doi: 10.1101/2023.01.12.523842 (PMC9882153; doi:10.1101/2023.01.12.523842)
Supplement: Supplementary file 1 [file media-1.pdf]

Supplemental Figure 1

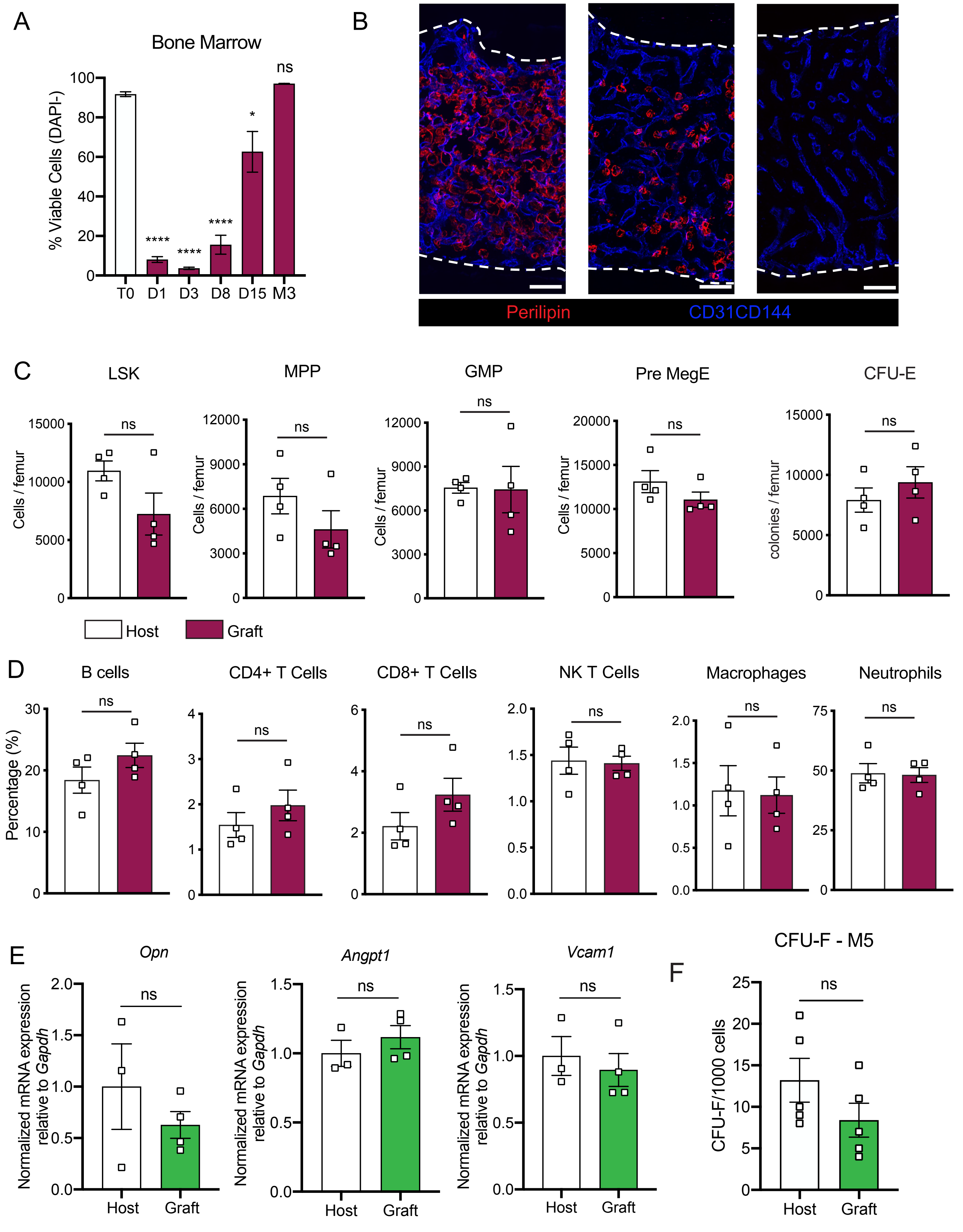

Supplemental Figure 1. Bone marrow regeneration after transplantation.

A. Cell viability determined by flow cytometry and expressed as percentage of total DAPI- alive cells in the BM graft at steady state, 1, 3, 8, 15 days and 3 months after bone transplantation (n=3-4). One-way ANOVA with Dunnett's multiple comparisons test used to determine statistical significance.
B. Representative whole-mount confocal z-stack projections of host and graft femurs at 1 and 4 months after transplantation. Adipocytes are stained using an anti-perilipin antibody. Scale bar = 50μm. Three independent experiments yielded similar results.
C. Comparison of different hematopoietic progenitors' populations between graft and host at 5 months after bone transplantation (n=4 per group).
D. Comparison of different hematopoietic populations between graft and host at 5 months after bone transplantation (n=4 per group).
E. Quantitative RT-PCR analysis of mRNA expression of *Opn*, *Angpt1*, and *Vcam1* relative to *Gapdh* in host and graft *Nes*-GFP^{+} BM-MSCs at 5 months after transplantation (n=3-4 mice).
F. Number of CFU-F colonies formed by flushed CD45^{-}Ter119^{-}*Nes*-GFP^{+} BM-MSCs sorted from host and graft femurs 5 months after transplantation and plated at equal number and clonal densities under CFU-F culture conditions (n=5 mice per group). Data represented as mean ± SEM. Unless otherwise noted, statistical significance was determined using unpaired two-tailed Student's t test. *p<0.05. ** p<0.01. *** p<0.001. ****p<0.0001.

Supplemental Figure 2

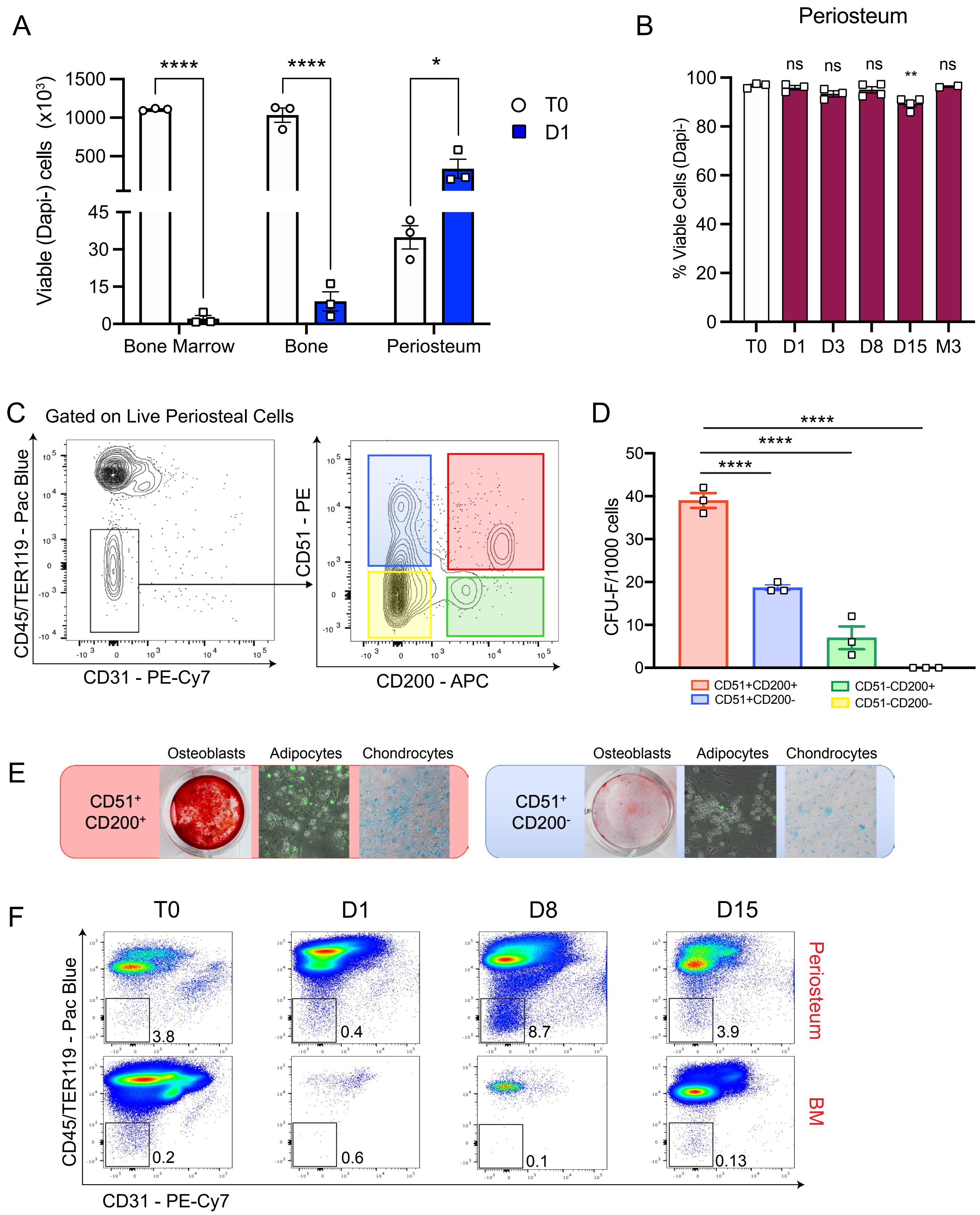

Supplemental Figure 2. Periosteal stem cells expansion after transplantation.

A. Number of viable cells within bone marrow, bone and periosteum at steady state and 24 hours post transplantation (n=3 per group).
B. Cell viability determined by flow cytometry and expressed as percentage of total DAPI- alive cells in the periosteum graft at steady state and at 1, 3, 8, 15 days and at 3 months after bone transplantation (n=2-4 per group). One-way ANOVA with Dunnett's multiple comparisons test was used to determine statistical significance.
C. Representative FACS plot showing gating of $CD51^{+}CD200^{+}$ P-SSCs after gating on stromal ($CD45^{-}Ter119^{-}CD31^{-}$) fraction.
D. Absolute CFU-F number from 1000 sorted cells within various cell populations of the periosteum. One-way ANOVA with Dunnett's multiple comparisons test was used to determine statistical significance.
E. Representative trilineage differentiation pictures of $CD51^{+}CD200^{+}$ compared to the group with the next highest CFU-F activity ($CD51^{+}CD200^{-}$ cells). Differentiation into osteoblasts, adipocytes and chondrocytes observed using Alizarin Red, Bodipy, and Alican Blue staining, respectively.
F. Representative FACS plots of graft periosteum and bone marrow analysis gated on CD45-Ter119-CD31- fraction at steady state and at one-, eight- and fifteen-days post transplantation. Data represented as mean ± SEM. Unless otherwise noted, statistical significance was determined using unpaired two-tailed Student's t test. *p<0.05. ** p<0.01. *** p<0.001. ****p<0.0001.

Supplemental Figure 3

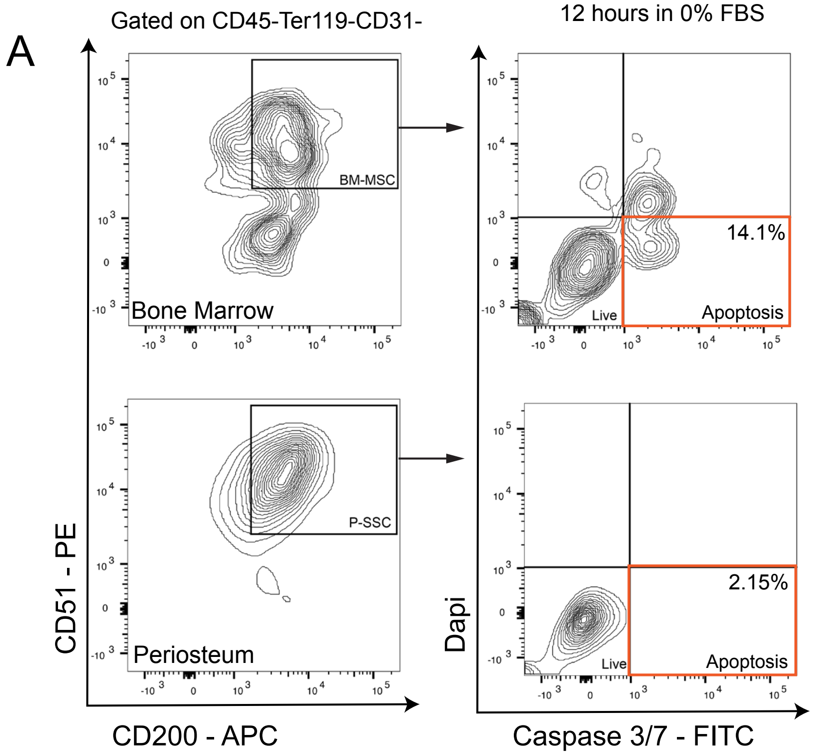

Supplemental Figure 3. Comparison of periosteal SSCs and bone marrow MSCs.

A. Representative FACS plots showing gating strategy for analyzing apoptosis at12 hours after switching from 20% to 0% FBS media.

Data represented as mean ± SEM. Unless otherwise noted, statistical significance was determined using unpaired two-tailed Student's t test. *p<0.05. ** p<0.01. *** p<0.001. ****p<0.0001.

Supplemental Figure 4

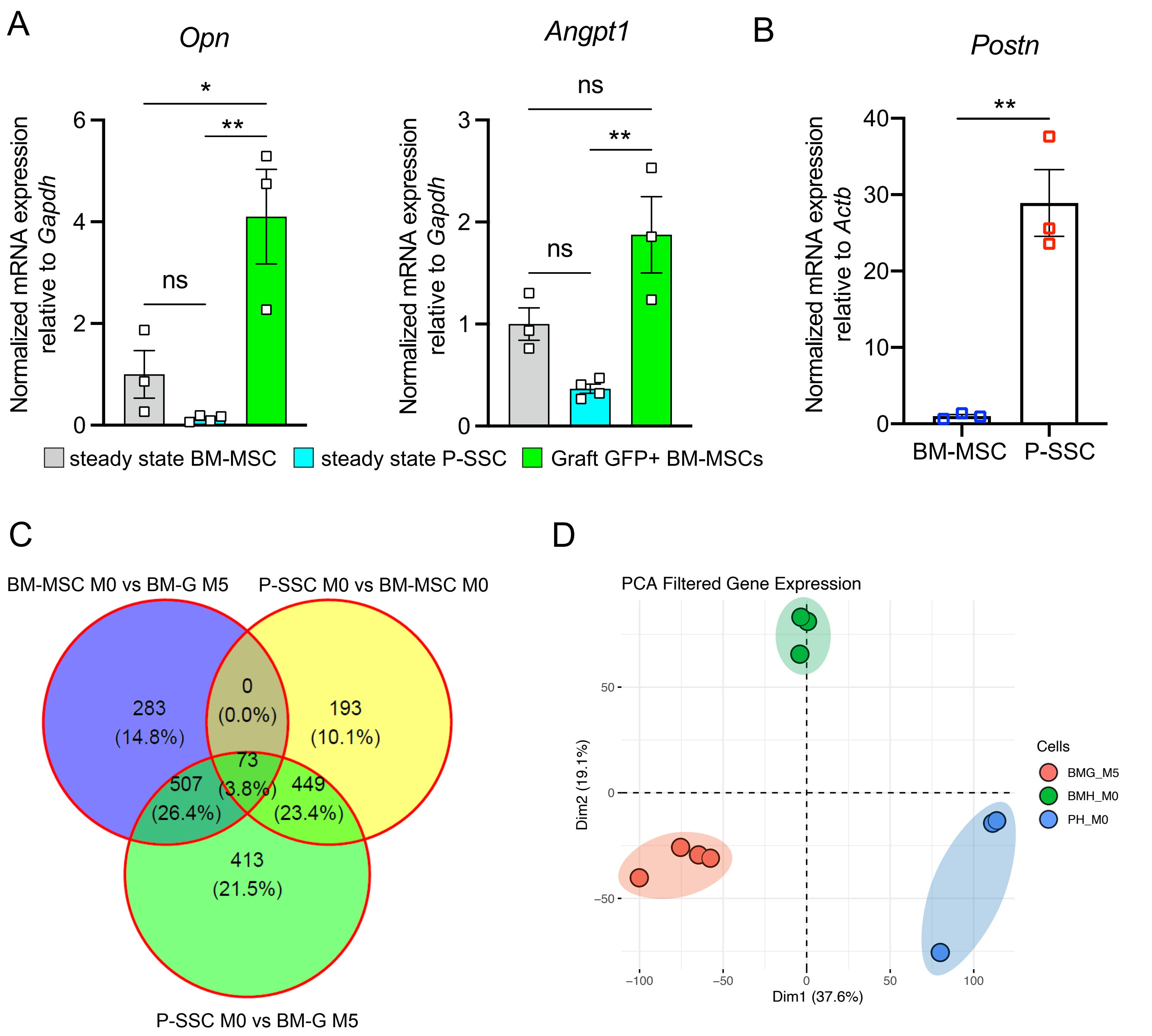

Supplemental Figure 4. Periosteal SSCs migrate into the bone marrow and upregulate HSC-niche factors.

A. Quantification of *Opn* and *Angpt1* mRNA level relative to *Gapdh* in sorted control bone marrow $CD45^{-}Ter119^{-}CD31^{-}Nestin\text{-}GFP^{+}$ BM-MSCs, $CD45^{-}Ter119^{-}CD31^{-}CD51^{+}CD200^{+}$ steady-state P-SSCs, and $CD45^{-}Ter119^{-}CD31^{-}CD51^{+}CD200^{+}GFP^{+}$ graft BM-MSCs (n=3-4 per group). One-way ANOVA was used to determine statistical significance.
B. *Postn* expression in sorted, steady-state $CD51^{+}CD200^{+}$ BM-MSCs and P-SSCs from CD45.2 WT mice (n=3).
C. Venn diagram of RNA sequencing analysis comparing the numbers of differentially expressed genes between the groups
D. 2D principal component analysis (PCA) plot showing the variance between graft BM-MSCs at five months post transplantation, and BM-MSCs and P-SSCs at steady state (n=3-4).

Data represented as mean ± SEM. Unless otherwise noted, statistical significance was determined using unpaired two-tailed Student's t test. *p<0.05. ** p<0.01. *** p<0.001. ****p<0.0001.

Supplementary Table 1: Flow Cytometry Antibodies

Antibody	Clone	Fluorochrome (Manufacturer)
anti-CD45	30-F11	APC-Cy7 (eBioscience), Pacific Blue (Biolegend)
anti-Ter119	Ter-119	APC-Cy7 (eBioscience), Pacific Blue (Biolegend), AF700 (Biolegend), biotin (BD Biosciences)
anti-CD31	390	PE-Cy7 (Biolegend; eBioscience), AF647 (Biolegend)
anti-CD51	RMV-7	biotin (Biolegend), PE (eBioscience)
anti-CD200	OX-90	APC (Biolegend)
anti-CD140α	APA5	PE-Cy7 (eBioscience), APC (Biolegend)
anti-strepavidin	(n/a)	FITC (eBioscience), APC-Cy7 (eBioscience), BV570 (Biolegend)
anti-CD11b	M1/70	AF647 (Biolegend), biotin (BD Biosciences)
anti-CD4	GK1.5	PE-Cy7 (eBioscience)
anti-CD8a	53-6.7	PE-Cy7 (eBioscience)
anti-B220	RA3-6B2	PerCP-Cy5.5 (eBioscience), biotin (BD Biosciences)
anti-CD45.1	A20	PE (Biolegend)
anti-CD45.2	104	FITC (Biolegend)
anti-Ki67	SolA15	FITC (eBioscience)
anti-CD144	BV13	AF647 (Biolegend)
anti-Gr-1	RB6-8C5	APC-Cy7 (eBioscience), FITC, biotin (BD Biosciences)
anti-CD150	TC15-12F12.2	PE (Biolegend)
anti-CD117	2B8	BV421 (Biolegend)
anti-Sca-1	D7	FITC (eBioscience)
anti-CD105	MJ7/18	PE-Cy7 (Biolegend)
anti-CD41	MWReg30	PerCP-e710 (eBioscience)
anti-CD16/32	93	APC-Cy7(Biolegend)
anti-CD48	HM48-1	AF647 (Biolegend)
anti-CD3e	145-2C11	biotin (BD Biosciences)
anti-F4/80	BM8	AF647 (Biolegend)
anti-NK1.1	PK136	BV421 (Biolegend)
anti-CD115	AFS98	PE (eBioscience)

Supplementary Table 2: RT-PCR primer sequences

**Primer**	**Forward Sequence (5' → 3')**	**Reverse Sequence (5' → 3')**
Cxcl12	5'-CGCCAAGGTCGTCGCCG-3'	5'-TTGGCTCTGGCGATGTGGC-3'
Kitl	5'-CCCTGAAGACTCGGGCCTA-3'	5'-CAATTACAAGCGAAATGAGAGCC-3'
Opn	5'-AGCAAGAAACTCTTCCAAGCAA-3'	5'-GTGAGATTCGTCAGATTCATCCG-3'
Angpt1	5'-CTCGTCAGACATTCATCATCCAG-3'	5'-CACCTTCTTTAGTGCAAAGGCT-3'
Vcam1	5'-GACCTGTTCCAGCGAGGGTCTA-3'	5'-CTTCCATCCTCATAGCAATTAAGGTG-3'
Cdkn1a	5' CCTGGTGATGTCCGACCTG-3'	5' CCATGAGCGCATCGCAATC-3'
Cdkn1c	5-CGAGGAGCAGGACGAGAATC-3'	5' GAAGAAGTCGTTCGCATTGGC-3'
Cdk4	5'-ATGGCTGCCACTCGATATGAA 3'	5' TCCTCCATTAGGAACTCTCACAC-3'
Sod1	5'-TGGTGGTCCATGAGAAACAA-3'	5'-GTTTACTGCGCAATCCCAAT-3'
Gls	5' TTCGCCCTCGGAGATCCTAC-3'	5' CCAAGCTAGGTAACAGACCCT-3'
Gpx1	5'-AGTCCACCGTGTATGCCTTCT-3'	5'- GAGACGCGACATTCTCAATGA-3'-3'
Postn	5'-CCTGCCCTTATATGCTCTGCT-3'	5'-AAACATGGTCAATAGGCATCACT-3'
Gapdh	5'-TGTGTCCGTCGTGGATCTGA-3'	5'-CCTGCTTCACCACCTTCTTGA-3'
Actb	5' GCTTCTTTGCAGCTCCTTCGT-3'	5' ATCGTCATCCATGCCGAACT-3'
